# Licensing Examination and Crash Outcomes Postlicensure in Young Drivers

**DOI:** 10.1001/jamanetworkopen.2022.8780

**Published:** 2022-04-25

**Authors:** Elizabeth A. Walshe, Daniel Romer, Abraham J. Wyner, Shukai Cheng, Michael R. Elliott, Robert Zhang, Alexander K. Gonzalez, Natalie Oppenheimer, Flaura K. Winston

**Affiliations:** 1 Center for Injury Research and Prevention, Children's Hospital of Philadelphia Research Institute, Children’s Hospital of Philadelphia, Philadelphia, Pennsylvania; 2 Annenberg Public Policy Center, University of Pennsylvania, Philadelphia; 3 Wharton School, University of Pennsylvania, Philadelphia; 4 School of Public Health, University of Michigan, Ann Arbor; 5 Institute for Social Research, University of Michigan, Ann Arbor; 6 Department of Biomedical and Health Informatics, Children's Hospital of Philadelphia Research Institute, Philadelphia; 7 Division of General Pediatrics, University of Pennsylvania Perelman School of Medicine; Philadelphia

## Abstract

**Importance:**

Despite US graduated driver licensing laws, young novice driver crash rates remain high. Study findings suggest comprehensive license policy that mandates driver education including behind-the-wheel (BTW) training may reduce crashes postlicensure. However, only 15 states mandate BTW training.

**Objective:**

To identify differences in licensing and crash outcomes for drivers younger than 18 years who are subject to comprehensive licensing requirements (graduated driver licensing, driver education, and BTW training) vs those aged 18 to 24 years who are exempt from these requirements.

**Design, Setting, and Participants:**

This prospective, population-based cohort study used Ohio licensing data to define a cohort of 2018 license applicants (age 16-24 years, n = 136 643) and tracked licensed driver (n = 129 897) crash outcomes up to 12 months postlicensure. The study was conducted from January 1, 2018, to December 31, 2019, and data analysis was performed from October 7, 2019, to February 11, 2022.

**Main Outcomes and Measures:**

Licensing examination performance and population-based, police-reported crash rates in the first 2 months and 12 months postlicensure across age groups, sex, and census tract–level sociodemographic variables were measured. Poisson regression models compared newly licensed driver crash rates, with reference to individuals licensed at 18 years, while controlling for census tract–level sociodemographic factors, time spent in the learner permit period, and licensing examination performance measures.

**Results:**

Of 136 643 novice drivers, 69 488 (50.9%) were male and 67 152 (49.1%) were female. Mean (SD) age at enrollment (age at first on-road examination) was 17.7 (2.1) years. License applicants aged 16 and 17 years performed best on license examinations (15 466 [21.6%] and 5112 [30.9%] failing vs 7981 [37.5%] of applicants aged 18 years). Drivers licensed at 18 years had the highest crash rates of all those younger than 25 years. Compared with drivers licensed at 18 years, crash rates were 27% lower in individuals aged 16 years and 14% lower in those aged 17 years during the first 2 months postlicensure when controlling for socioeconomic status, time spent in learner permit status, and license examination performance measures (adjusted relative risk [aRR] at age 16 years: 0.73; 95% CI, 0.67-0.80; age 17 years: aRR, 0.86; 95% CI, 0.77-0.96). At 12 months postlicensure, crash rates were 19% lower for individuals licensed at age 16 years (aRR, 0.81; 95%, CI, 0.77-0.85) and 6% lower at age 17 years (aRR, 0.94; 95% CI, 0.89-0.99) compared with individuals aged 18 years.

**Conclusions and Relevance:**

In Ohio, drivers younger than 18 years who are subject to graduated driver licensing and driver education, including BTW training requirements, had lower crash rates in the first year postlicensure compared with those aged 18 years, with controls applied. These findings suggest that it may be fruitful for future work to reconsider the value of mandated driver license policies, including BTW training, and to examine reasons for delayed licensure and barriers to accessing training.

## Key Points

**Question:**

Do drivers younger than 18 years and subject to comprehensive licensing policy (graduated driver licensing laws, mandatory driver education and training) have better licensing and crash outcomes compared with drivers aged 18 to 24 years who are exempt from these licensing policies?

**Findings:**

In this cohort study of 136 643 individuals aged 16 to 24 years, license applicants aged 16 to 17 years performed better on license examinations than those aged 18 to 24 years. Individuals licensed when younger than 18 years had lower crash rates in the first year of licensure than those licensed at 18 years, and individuals licensed at 18 years had the highest crash rates of all those younger than 25 years.

**Meaning:**

The findings of this study suggest that it may be useful to reevaluate comprehensive driver licensing policies, including driver training, as a strategy to reduce crashes in young novice drivers.

## Teaser

This cohort study examines licensure outcomes and crash rates in individuals aged 18 to 24 years according to mandated driver education.

## Introduction

Throughout the US, graduated driver licensing (GDL) laws have contributed to lowering motor vehicle crash risk^1^; however, crash rates (both per driver and per mile driven) remain disproportionately high among young, novice drivers.^2,3,4^ Motor vehicle crashes are one of the most preventable public health problems in the US: driver error accounts for an estimated 96% of crashes,^5^ and newly licensed drivers commit more safety-critical driving errors than more experienced drivers.^6^

Graduated driver licensing safety gains have been associated with reduced exposure through delayed licensure and restricted driving (eg, nighttime restrictions).^1,7^ However, Lyon et al^8^ found variability in GDL benefits across states: those with strict laws (longer time in learner permit [TLP] period and driver training requirements for adolescents aged 16 years) reported lower crash rates. Most states, however, have relatively lenient GDL laws, requiring only classroom lessons and supervised practice behind the wheel (BTW), in addition to GDL, for those younger than 18 years. Only 15 states have more comprehensive policies that mandate BTW training with a professional instructor.^9^

The effectiveness of BTW training in reducing crashes was questioned based on a 1983 trial in DeKalb, Georgia,^10,11,12^ which predated GDL implementation and current driver training standards. Initial and repeated analyses of the DeKalb data noted a protective result associated with training on 6-month crash outcomes (reduced by 13.1% in a 2011 analysis)^12^ when the burden of crashes is highest for novice drivers.^4,13,14^ However, the lack of persistent crash reduction benefits at 1- to 2-year follow-up^11,12,15,16^ was used as an argument against mandating BTW training as part of licensing policy. Furthermore, it has been argued that any early-in-licensure crash reduction could be offset by more young drivers becoming licensed earlier (a benefit of completing BTW training), and thus an overall increased burden of crashes due to the greater number of the highest-risk drivers.^12^

However, California data from 2000 to 2007 show that drivers younger than 18 years and subject to comprehensive licensing requirements (including BTW training) have lower crash rates, especially in the early months of licensure, than those licensed at 18 years who are required to have only a 24-hour permit holding period and no BTW training.^17^ Studies in Nebraska, Oregon, and Manitoba, Canada,^18,19^ also suggest that licensing policies including BTW training are associated with reduced crashes postlicensure. However, none of these studies was able to determine the relative contribution of minimum TLP periods, BTW training, or delayed licensure among those at increased risk of crashing (eg, lower socioeconomic status^20,21^) to reductions in crash rates.

Therefore, using an existing partnership with Ohio,^22^ we conducted a large, population-based, prospective cohort study to test the hypothesis that comprehensive licensing policy that mandates BTW training in addition to GDL is associated with reducing the heightened age-related crash burden for drivers younger than 18 years. To do this, we linked 2018 statewide licensing data and subsequent police-reported crash records up to 2 and 12 months postlicensure to quantify crash outcomes by age in newly licensed drivers aged 16 to 24 years, while controlling for sex, census tract–level sociodemographic indicators, and approximations of skill and experience at the time of licensure (TLP status, final license examination score, and number of failed licensing examinations). Including sociodemographic confounding factors has been reported to overcome potential bias in previous state-based studies; in particular, household income is a known risk for crashes^23,24^ and delayed licensure.^20^

We hypothesized that, compared with drivers licensed at age 18 years, those licensed when younger than 18 years with driver education and BTW training in addition to GDL will have better skills, suggested by fewer of them failing the licensing examination and no greater involvement in crashes early in licensure (0-2 months) and over the full first year postlicensure. Providing data that track an individual through 12 months postlicensure offers an opportunity to observe the outcomes associated with enhanced GDL requirements for reducing the population burden of crashes in the first year of driving.

## Methods

### Ohio Licensing Policy

In addition to typical GDL restrictions, Ohio license applicants younger than 18 years are required to (1) complete 24 hours of classroom or online instruction; (2) complete 8 hours of BTW training at a licensed driving school; (3) complete 50 hours of practice driving, including 10 hours of night driving; and (4) hold the temporary permit for at least 6 months. When applicants become aged 18 years, they are not subject to any of the above before attempting the road safety examination (RSE). The RSE in Ohio is a 2-part test: a maneuverability test that requires the applicant to demonstrate basic control by steering the vehicle around markers and a driving skills test that assesses the applicant’s ability to handle turns, starts and stops, reverses, signal use, lane choice, and success in maintaining safe following distances from other vehicles. Drivers must have an error score less than 26 to pass each test. The study was conducted from January 1, 2018, to December 31, 2019, and data analysis was performed from October 7, 2019, to February 11, 2022. The study was exempted from institutional review board oversight by Children’s Hospital of Philadelphia owing to use of deidentified data. This study followed the Strengthening the Reporting of Observational Studies in Epidemiology (STROBE) reporting guideline for cohort studies.

### Data Sources: Ohio Licensing and Crash Databases

This investigation used 2 statewide secure, confidential databases maintained by the Ohio Department of Public Safety: the Ohio Bureau of Motor Vehicles licensing database and the Ohio Department of Public Safety crash database. The licensing database contains detailed information on each driver’s interactions with the Ohio Bureau of Motor Vehicles, including driver demographics (date of birth, sex, and address), RSE scores and outcomes (pass and fail), licensing transaction dates, and a completed training date. Sex responses include male, female, and unknown (accounted for 0.04%). Ohio crash data are collected from more than 1000 law enforcement agencies via a statewide uniform crash report that was updated in 2011 and is in compliance with the current edition of Model Minimum Uniform Crash Criteria standards.^25^ In Ohio, a crash must be reported if any personal injury or fatality occurs and/or there is at least $1000 worth of property damage.

### Data Set Preparation

**Figure 1.**
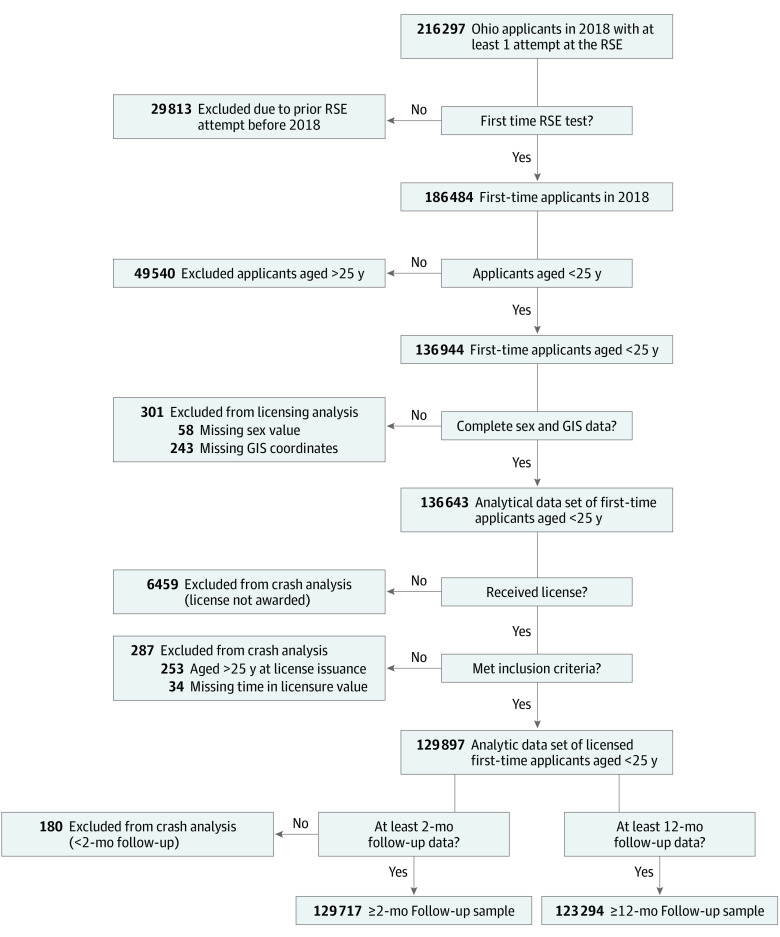
Sample Derivation for RSE Outcome Analysis and Crash Outcome Analysis GIS indicates geographical information system; RSE, road safety examination.

A data operations team at the Children’s Hospital of Philadelphia prepared a linked, deidentified analytic data set in accordance with data privacy agreements between Ohio and the Children’s Hospital of Philadelphia. In brief, licensing records were merged with the following variables from various licensing database tables: permit issue date, date of RSE attempt, license issue date, sex, training completion date, and RSE test scores and outcomes (pass and fail). All data were merged via primary keys and common identifiers in each source table. Then, the licensing data were merged with the crash database for 2018 and 2019, using driver license number to match records. Licensing data from the full year of 2018 were used to avoid any seasonality effects and allow a full year of follow-up for drivers who were licensed in 2018. All dates were converted to age in days from birthdate including age at permit issuance, RSE attempt, license issuance, and any crash reported. From these data, we derived TLP, time since licensure, and time to first crash. In this sample, 98 861 (72.4%) first-time license applicants had only 1 learner permit before attempting the RSE. A binary completed training (yes/no) variable was derived from training completion date. Driver license addresses were matched to Federal Information Processing System codes using a geocoder (ArcGIS, Esri), and the Federal Information Processing System codes were matched to the American Community Survey, using 5-year estimates from 2015 to 2019^26^ to extract census tract–level sociodemographic variables. A small percentage of drivers (0.18%) in our sample had missing sociodemographic information because not all addresses could be geocoded (Figure 1).

A cohort of license applicants was created by including those younger than 25 years at their first attempt at the RSE in 2018. Those with earlier exposure to the licensing process (eg, expired or suspended licenses or failed attempts at the RSE) were excluded (Figure 1). The final analytic data set comprised 136 643 individuals. Approximately 95% of the applicants were issued a license and thus were retained for the crash analysis. We analyzed 129 717 drivers for whom we had at least 2-month follow-up postlicensure data and 123 294 drivers for whom we had at least 12-month follow-up postlicensure data. Only postlicensure crashes were examined for this analysis, but a small number of applicants (1630 [1.2%]) had a police-reported crash before receiving a license.

### Outcomes

The RSE outcome was defined as the percentage of drivers who failed either the maneuverability or driving skills subtest on their first try. To examine population-level crash burden, we calculated the rate of crashes each month postlicensure for the following age groups: 16, 17, 18, 19 to 20, and 21 to 24 years (rounded to the nearest integer). The monthly rate of crashes was calculated as the number of police-reported crashes each month divided by the number of licensed drivers in each age group. The count of crashes for every driver during each period (0-2 or 0-12 months) served as the outcome in the estimation models.

For describing license examination outcomes, age in days at the first attempt of the RSE was used. When examining crash outcomes, age in years at licensure was used as a proxy of the licensing policy: only 0.8% of applicants who were aged 18 years or older at the time of their first license application had completed driver training. Sex was determined from entries for male and female. For drivers who received a license, TLP was determined as the time between learner permit issuance and license issuance, which has a maximum of 12 months. To control for driving skill, the number of earlier failed attempts of the RSE and the score on the RSE driving skill test from the final (passing) RSE that granted licensure were also included in the analyses.

Because individual-level identifying data were not available beyond age and sex, census tract–level variables were obtained from the American Community Survey^26^ based on home residence and represented domain 1, income and educational level (7 indicators); domain 2, transportation access and use patterns (8 indicators); and domain 3, urbanicity (4 indicators), as well as race and ethnicity. A principal component analysis of the 3 domains identified 6 components with eigenvalues greater than 0.9 that were used to create summary scores for further analysis. Of the 3 domains and race and ethnicity, only 1 component that distinguished census tracts—household income and higher educational level (hereafter referred to as SES)—remained associated with crash outcomes and was the only American Community Survey–derived variable included in subsequent models.

### Statistical Analysis

Stepwise Poisson regression models were used to estimate the relative risks associated with age in estimating crash counts in the 0- to 2-month and 0- to 12-month follow-up periods. For all models, we used the age 18-year group as the reference for comparison. The model building process began with including age, the SES component described above (household income and higher educational level), and the licensing performance variables (TLP, number of failed RSE attempts, and final RSE driving subtest score). Interactions between the discrete variables were also considered. Ultimately, only age, the SES component (household income and educational level), and licensing performance variables were consistently and reliably found to estimate crash outcomes over 0 to 2 and 0 to 12 months. The subsequent analysis was conducted in 3 steps, with age entered first, the SES component second, and the licensing history variables third. To account for overdispersion of crash counts, a scaled variance estimator^27^ was used, although the scale factor was very close to 1 in all analyses, suggesting little overdispersion. All analyses were conducted in SAS, version 9.4, and JMP, version 16 (SAS Institute Inc). We used a 2-sided α level of .05 to determine significance; all data are unpaired.

## Results

Table 1 presents the characteristics of the sample of all statewide 2018 first-time RSE applicants and the subset of those who were issued a license. Of 136 643 individuals younger than 25 years, most (71 490 [52.3%]) were age 16 years, with smaller representation in other age groups and approximately equal male (69 488 [50.9%]) and female (67 152 [49.1%]) distribution. Mean (SD) age at enrollment (age at first on-road examination) was 17.7 (2.1) years.

**Table 1.** Characteristics of First-time Applicants^a^

Variable	No. (%)			Mean (SD)		
	Overall	Failed first RSE	Issued a license	No. of failed RSEs before licensure	Time with learner permit before licensure, d	Driving skill test score on final passing RSE
Age, y						
All <25	136 643	40 553 (29.7)	130 184 (95.3)	1.20 (0.50)	200 (101.21)	10.92 (8.16)
16	71 490 (52.3)	15 466 (21.6)	71 275 (99.7)	1.14 (0.41)	224 (72.53)	9.93 (7.97)
17	16 521 (12.1)	5112 (30.9)	16 267 (98.5)	1.22 (0.53)	206 (111.36)	11.07 (8.12)
18	21 301 (15.6)	7981 (37.5)	19 466 (91.4)	1.26 (0.56)	160 (119.44)	12.42 (8.19)
19-20	14 647 (10.7)	6477 (44.2)	12 521 (85.5)	1.33 (0.65)	169 (124.87)	12.85 (8.22)
21-24	12 684 (9.3)	5517 (43.5)	10 655 (84.0)	1.32 (0.66)	142 (124.84)	12.83 (8.23)
Sex^b^						
Male	69 488 (50.9)	19 434 (28.0)	66 432 (95.6)	1.20 (0.50)	195 (102.87)	10.98 (8.18)
Female	67 152 (49.1)	21 118 (31.4)	63 749 (94.9)	1.21 (0.51)	206 (99.11)	10.87 (8.14)
Tract-level SES: income and educational level						
Low: <10th percentile	13 670 (10.0)	5264 (38.5)	12 055 (88.2)	1.26 (0.58)	175.11 (118.03)	12.56 (8.14)
Middle: >10th-<90th percentile	109 339 (80.0)	31 825 (29.1)	104 713 (95.8)	1.20 (0.50)	201.48 (100.48)	10.96 (8.17)
High: >90th percentile	13 634 (10.0)	3464 (25.4)	13 416 (98.4)	1.19 (0.49)	212.96 (85.59)	9.62 (7.89)

Abbreviations: RSE, road safety examination; SES, socioeconomic status.

^a^ For descriptive purposes, a census tract–level categorization of the SES component is shown here, but the continuous component score was used in all subsequent model analyses.

^b^ Data missing on 3 individuals.

### Licensing Outcomes

At the population level, the youngest applicants at age 16 years (53.3%) and 17 years (12.1%) failed their first RSE the least of all age groups; had fewer failed attempts overall (16 years: 15 466 [21.6%]; 17 years: 5112 [30.9%]); and had fewer errors on their final passing RSE driving skill test, with the age 16-year group performing best (ie, lowest score) (mean [SD] score, 16 years: 9.93 [7.97]; 17 years: 11.07 [8.12]) (Table 1). Drivers licensed at age 18 years had the shortest (mean [SD], 160 [119.44] days) TLP compared with TLP of all those in the age 16- to 24-year sample (mean [SD], 200 [101.21] months). There were also differences in census tract–level SES, whereby those in lower income and educational level tracts were more likely to fail their first RSE (<10th percentile: 5264 [38.5%] vs >90th percentile: 3464 [25.4%]).

### Crash Outcomes

Drivers licensed at ages 16 and 17 years had lower crash rates and those licensed at age 18 years had the highest crash rates per 1000 licensed drivers during the first 2 months (16 years: 26.0, 17 years: 32.5, 18 years: 40.6 months) and during the first 12 months (16 years: 129.7, 17 years: 158.3, 18 years: 179.9 months). Therefore, compared with drivers licensed at age 16 years, those licensed at 18 years had 14.6 additional crashes per 1000 licensed drivers at 2 months and 50.2 additional crashes per 1000 licensed drivers at 12 months (Table 2). Male (2 months: 29.4; 12 months: 143.1) and female (2 months: 29.7; 12 months: 142.7) drivers had comparable rates per 1000 licensed drivers. However, drivers living in census tracts representing the lowest 10th percentile of SES had approximately 18 additional crashes per 1000 drivers during the first 2 months (37.9) and 78 additional crashes per 1000 drivers during the first 12 months (176.1) postlicensure compared with the highest 10th percentile of SES (2 months: 20.1; 12 months: 98.4).

**Table 2.** Licensed Drivers and Total Number of Crashes per 1000 Licensed Drivers Postlicensure^a^

Variable	Follow-up			
	2 mo		12 mo	
	No. of licensed drivers	Total No. of crashes per 1000 licensed drivers	No. of licensed drivers	Total No. of crashes per 1000 licensed drivers
Age at licensure, y				
All <25	129 717	29.6	123 294	142.9
16	70 344	26.0	69 367	129.7
17	16 425	32.5	15 821	158.3
18	18 541	40.6	17 295	179.9
19-20	13 374	31.4	11 170	156.2
21-24	11 033	27.2	9641	130.5
Sex				
Male	66 185	29.4	63 024	143.1
Female	63 529	29.7	60 267	142.7
Census tract–level SES: income and education				
Low: <10th percentile	12 825	37.9	11 717	176.1
Middle: >10th to <90th percentile	103 988	29.7	99 092	144.6
High: >90th percentile	12 904	20.1	12 485	98.4

Abbreviation: SES, socioeconomic status.

^a^ For descriptive purposes, a census tract–level categorization of the SES component is shown here, but the continuous component score was used in all subsequent model analyses.

**Figure 2.**
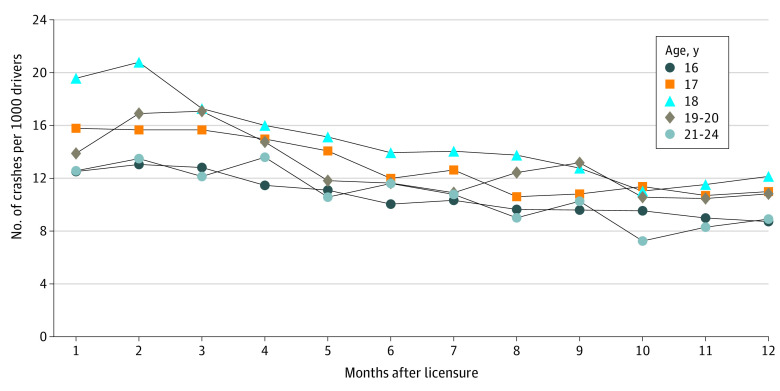
Monthly Crash Counts per 1000 Drivers in Each Age Group Over the First 12 Months Postlicensure The oldest and youngest age groups have nearly indistinguishable trajectories. The 18-year-olds have the highest crash rates over time.

When examining the population-based monthly crash rates for each age group during the first year of licensure (Figure 2) we noted a similar pattern: drivers licensed at age 18 years had the highest monthly crash rates per 1000 drivers than any other age group, with those aged 16 years and 21 to 24 years showing the lowest rates. The rates of drivers licensed at age 17 years were between the rates for those licensed at 16 and 18 years, overlapping with rates of those aged 19 to 20 years, which appear to be more variable, but the sample size was smaller. The difference in monthly crash rates between the age 16-, 17-, and 18-year groups was greatest over the first 2 months of licensure, and dissipated over the latter half of the year.

Poisson regression model analyses supported the association between age and crash rates over the first 2 months and 12 months postlicensure, even when controlling for census tract–level SES, and licensing variables (approximating skill and experience) that also were associated with crash rates (Table 3). Those licensed at age 18 years had significantly higher crash rates than any other age group. Compared with drivers licensed at 18 years, the crash rate during the first 2 months of licensure was 27% lower for those licensed at age 16 years (adjusted relative risk [aRR], 0.73; 95% CI, 0.67-0.80) and 14% lower for those licensed at age 17 years (aRR, 0.86; 95% CI, 0.77-0.96), when controlling for all covariates. Similarly, during the first 12 months postlicensure, the crash rate was 19% lower for individuals licensed at age 16 years (aRR, 0.81; 95% CI, 0.77-0.85) and 6% lower for those licensed at age 17 years (aRR, 0.94; 95% CI, 0.89-0.99) compared with those licensed at age 18 years. However, the relative differences in crash rates between the 18-year and younger age groups decreased when adding these covariates. Crash rates were 1% lower at 12 months and 2% lower at 2 months for each additional TLP month and 8% higher at 12 months and 11% higher at 2 months for each additional failed RSE attempt before licensure.

**Table 3.** Association of Age at Licensure With Crash Outcomes at 2 and 12 Months^a^

Metric	Outcome of age at licensure		Outcome of age at licensure controlling for SES		Outcome of age at licensure controlling for SES and licensing variables	
	Relative risk (95% CI)	*P* value	Adjusted relative risk (95% CI)	*P* value	Adjusted relative risk (95% CI)	*P* value
**2-Month model**						
Age at licensure, y						
16 vs 18	0.64 (0.59-0.70)	<.001	0.69 (0.63-0.75)	<.001	0.73 (0.67-0.80)	<.001
17 vs 18	0.80 (0.72-0.90)	<.001	0.83 (0.75-0.93)	.001	0.86 (0.77-0.96)	.009
19-20 vs 18	0.77 (0.69-0.87)	<.001	0.77 (0.68-0.87)	<.001	0.77 (0.68-0.87)	<.001
21-24 vs 18	0.67 (0.59-0.76)	<.001	0.67 (0.58-0.76)	<.001	0.65 (0.56-0.74)	<.001
Census tract–level SES: income and educational level (per unit change from average)	NA	NA	0.93 (0.92-0.95)	<.001	0.93 (0.92-0.95)	<.001
<10th percentile vs mean SES^b^	NA	NA	1.17 (1.13-1.21)	NA	1.17 (1.13-1.21)	NA
>90th percentile vs mean SES^b^	NA	NA	0.81 (0.77-0.85)	NA	0.81 (0.77-0.85)	NA
Time with learner permit, mo	NA	NA	NA	NA	0.98 (0.97-0.99)	<.001
No. of failed RSE attempts	NA	NA	NA	NA	1.11 (1.04-1.18)	.003
Final RSE score	NA	NA	NA	NA	1.01 (1.00-1.01)	.005
**12-Month model**						
Age at licensure, y						
16 vs 18	0.72 (0.69-0.75)	<.001	0.77 (0.74-0.81)	<.001	0.81 (0.77-0.85)	<.001
17 vs 18	0.88 (0.83-0.93)	<.001	0.91 (0.87-0.96)	<.001	0.94 (0.89-0.99)	.02
19-20 vs 18	0.87 (0.82-0.92)	<.001	0.86 (0.81-0.92)	<.001	0.87 (0.82-0.92)	<.001
21-24 vs 18	0.73 (0.68-0.78)	<.001	0.72 (0.67-0.77)	<.001	0.72 (0.68-0.77)	<.001
Census tract–level SES: income and educational level (per unit change from average)	NA	NA	0.94 (0.93-0.95)	<.001	0.94 (0.93-0.95)	<.001
<10th percentile vs mean SES^b^	NA	NA	1.15 (1.13-1.17)	NA	1.15 (1.13-1.17)	NA
>90th percentile vs mean SES^b^	NA	NA	0.82 (0.80-0.84)	NA	0.83 (0.81-0.85)	NA
Time with learner permit, mo	NA	NA	NA	NA	0.99 (0.98-0.99)	<.001
No. of failed RSE attempts	NA	NA	NA	NA	1.08 (1.05-1.12)	<.001
Final RSE score	NA	NA	NA	NA	1.00 (1.00-1.01)	<.001

Abbreviations: NA, not applicable; RSE, road safety examination; SES, socioeconomic status.

^a^ Individuals aged 18 years at licensure were used as the reference group. Poisson model results with stepwise controls for SES and license variables.

^b^ Shown as an example to aid in interpretation of SES component score, which was included as a continuous variable in the model.

## Discussion

To our knowledge, this is the first population-level analysis to examine licensing and police-reported crash outcomes postlicensure as 2 characteristics of young drivers’ preparation for independent driving, including licensing performance and SES covariates, in a state that mandates driver education, including BTW training for those younger than 18 years, in addition to GDL. In contrast to earlier studies of age-related crash rates in which the younger novice drivers were reported to have the highest crash rates,^13,28^ our study noted that drivers licensed at age 18 years had the highest crash rates of all those in the age 16- to 24-year sample. Drivers licensed at age 16 to 17 years had crash rates 6% to 19% lower than those aged 18 years during 12 months postlicensure, and 14% to 27% lower during the first 2 months postlicensure, even when controlling for census tract–level SES and measures of driving skill (TLP status, number of RSE failures, and passing RSE driving score). License applicants aged 16 and 17 years also performed best on license examinations.

The finding that the association between age and outcomes for drivers aged 16 to 17 years decreased when indicators of SES, driving experience, and skill were added to the model appears to add evidence that those factors may be associated with the age differences observed. The association between low income and increased incidence of negative outcomes may be related to the fairly substantial costs associated with training, testing, and issuance of the license in Ohio ($400-$550 for instruction, BTW training, permit, and license).^29^ Thus, these findings suggest a potential benefit from 2 elements of Ohio’s comprehensive licensing policy on young driver crash burden: mandatory learner permit holding period (TLP tended be longer in drivers <18 years) and required training (less RSE failure and improved scores suggesting greater skills).

Our findings in Ohio, along with those reported from California,^17^ which also mandates BTW training as part of driver education, suggest that reexamination of comprehensive driver education that includes BTW training may be a strategy to further reduce the crash burden of the youngest drivers. Further study is warranted to examine the effect of BTW training on crash outcomes while accounting for exposure as a confounding factor. However, these findings also suggest that any requirement to obtain professional BTW training at or beyond age 18 years could place a burden on drivers from lower SES census tracts that would reduce their ability to drive. Need-based reductions in tuition for driver training may be needed to increase equity in policies designed to increase driver safety without also harming access to transportation for people with lower income levels.

### Limitations

This study has limitations. These data did not allow for a direct comparison of applicants aged 18 years and older with and without driver training because almost no applicants in that category completed BTW training before licensure. In addition, we did not have exposure data to examine crashes relative to the number of miles driven within each age group and must consider the age-related GDL restrictions in place. We also were limited to census tract–level sociodemographic confounding factors rather than individual-level indicators, and consideration of this lack of data may be relevant in interpretation of the findings.

## Conclusions

This cohort study found that, in Ohio, a state that mandates GDL and driver education including BTW training for license applicants younger than 18 years, drivers licensed at age 18 years exhibited the highest population-based rates of crashes in the first 2 and 12 months postlicensure, with those aged 16 and 17 years demonstrating significantly lower crash rates. Thus, Ohio’s driver license policies appear to be associated with a reduced incidence of crashes for the most at-risk new drivers: those licensed when younger than 18 years. It may be useful for future work to test the effects of mandated driver training for individuals up to age 18 years in a trial that controls for driving exposure to inform interventions and advances in driver training.
